# Work Attendance with Acute Respiratory Illness Before and During COVID-19 Pandemic, United States, 2018–2022

**DOI:** 10.3201/eid2912.231070

**Published:** 2023-12

**Authors:** Faruque Ahmed, Mary Patricia Nowalk, Richard K. Zimmerman, Todd Bear, Carlos G. Grijalva, H. Keipp Talbot, Ana Florea, Sara Y. Tartof, Manjusha Gaglani, Michael Smith, Huong Q. McLean, Jennifer P. King, Emily T. Martin, Arnold S. Monto, C. Hallie Phillips, Karen J. Wernli, Brendan Flannery, Jessie R. Chung, Amra Uzicanin

**Affiliations:** Centers for Disease Control and Prevention, Atlanta, Georgia, USA (F. Ahmed, B. Flannery, J.R. Chung, A. Uzicanin);; University of Pittsburgh, Pittsburgh, Pennsylvania, USA (M.P. Nowalk, R.K. Zimmerman, T. Bear);; Vanderbilt University Medical Center, Nashville, Tennessee, USA (C.G. Grijalva, H.K. Talbot);; Kaiser Permanente Southern California, Pasadena, California, USA (A. Florea, S.Y. Tartof);; Texas A&M University College of Medicine, Temple (M. Gaglani);; Baylor Scott and White Health, Temple, Texas, USA (M. Gaglani, M. Smith);; Marshfield Clinic Research Institute, Marshfield, Wisconsin, USA (H.Q. McLean, J.P. King);; University of Michigan, Ann Arbor, Michigan, USA (E.T. Martin, A.S. Monto);; Kaiser Permanente Washington Health Research Institute, Seattle, Washington, USA (C.H. Phillips, K.J. Wernli)

**Keywords:** coronavirus disease, severe acute respiratory syndrome coronavirus 2, COVID-19, SARS-COV-2, acute respiratory illness, respiratory infections, viruses, influenza, pandemics, presenteeism, teleworking, disease transmission, workplaces, United States

## Abstract

Both SARS-CoV-2 and influenza virus can be transmitted by asymptomatic, presymptomatic, or symptomatic infected persons. We assessed effects on work attendance while ill before and during the COVID-19 pandemic in the United States by analyzing data collected prospectively from persons with acute respiratory illnesses enrolled in a multistate study during 2018–2022. Persons with previous hybrid work experience were significantly less likely to work onsite on the day before through the first 3 days of illness than those without that experience, an effect more pronounced during the COVID-19 pandemic than during prepandemic influenza seasons. Persons with influenza or COVID-19 were significantly less likely to work onsite than persons with other acute respiratory illnesses. Among persons with positive COVID-19 test results available by the second or third day of illness, few worked onsite. Hybrid and remote work policies might reduce workplace exposures and help reduce spread of respiratory viruses.

COVID-19 cases in the United States, first reported on January 22, 2020, began to increase in March 2020 (*1*). The pandemic resulted in a substantial number of employed persons being laid off or furloughed, especially during spring 2020, and increased prevalence of teleworking (*2*–*4*). Employers were advised to actively encourage employees with symptoms of any acute respiratory illness (ARI) to stay home (*5*). Both SARS-CoV-2 and influenza viruses can be transmitted by infected persons who are asymptomatic, presymptomatic, or symptomatic (*6*,*7*); staying home while ill can reduce workplace virus transmission by reducing contacts between infectious and healthy persons (*8*). That policy is considered an everyday preventive action that should be implemented year-round, but especially during annual seasonal influenza seasons and pandemics (*9*).

Data collected during the early COVID-19 pandemic (March 26, 2020–November 5, 2020) showed that employed adults with previous telework experience were less likely than those without to work at the worksite (onsite) while sick (*10*). However, whether persons worked onsite within the early days of illness when infectiousness is higher has remained unclear (*7*,*11*,*12*). We aimed to assess the effects before and during the COVID-19 pandemic of employees’ previous experience with various work-location practices on work attendance patterns within the first 3 days of illness among persons with any ARI, including COVID-19 and influenza. Institutional review boards at the Centers for Disease Control and Prevention and all participating sites approved the study. The enrollees provided informed consent. 

## Methods 

### Study Population 

During November 12, 2018–June 30, 2022, the US Influenza Vaccine Effectiveness Network enrolled adults 19–64 years of age from network-affiliated sites in 7 states. During November 12, 2018–March 18, 2020, persons seeking care for an ARI with cough within 7 days of illness onset were enrolled after local influenza circulation was identified from outpatient facilities affiliated with network sites in 5 states: Michigan (Ann Arbor and Detroit); Pennsylvania (Pittsburgh); Texas (Temple and surrounding areas in central Texas); Washington (Puget Sound region); and Wisconsin (Marshfield, Wausau, and Weston). For the period October 14, 2020–June 30, 2022, case definition was broadened to include persons seeking treatment at outpatient or telehealth facilities within 10 days of illness onset with cough, fever, loss of taste or smell, or seeking clinical COVID-19 testing. Two additional sites, southern California region and Nashville, Tennessee, participated during October 2021–June 2022. For our study, we considered November 2018–March 2020 the period of prepandemic influenza seasons and October 2020–June 2022 the COVID-19 pandemic period. Detailed study methods have been published elsewhere (*13*–*15*). 

### Data Collection

Data were collected from patients at enrollment throughout the entire study period (November 2018–June 2022): date of illness onset, symptoms since illness began (including fever/feverishness), age, sex, race/ethnicity, education, self-rated general health status, cigarette smoking, and number of children <12 years of age living in household. Respiratory specimens were collected from all participants at enrollment and tested for influenza viruses using real-time reverse transcription PCR (rRT-PCR); during the COVID-19 period (2020–2022), specimens were also tested for SARS-CoV-2 using RT-PCR. Persons enrolled on or after January 15, 2022, were asked if they had taken an at-home rapid COVID-19 test while ill and whether the result was positive. 

All participants were asked to complete a follow-up survey, either online or over the phone, 1–2 weeks after enrollment. Throughout the 4-year study period, participants were asked at follow-up whether they had fully or mostly recovered from their illness and about employment status, type of employment (hourly, salaried, or other), hours they expected to work and hours usually worked from home in a typical week, and whether the employer discouraged workers with influenza-like symptoms from coming to work (Appendix Table 1). They were also asked if and where they worked on each of the first 3 days of illness (the first day being the day that symptoms started). Participants were asked about work status for the day before illness onset during November 2018–May 2019 at the Pennsylvania site and at all participating sites for the subsequent study years (Appendix Table 2). For the period November 2018–September 2021, two sites, in Washington and Wisconsin, did not collect data about work status while ill from participants who typically worked remotely before illness onset. For prepandemic influenza seasons, participants were asked at follow-up whether they worked in a healthcare setting with direct patient contact; that question was asked at enrollment during the COVID-19 pandemic period. 

### Definitions 

To categorize work experience before illness onset for our study, we used responses to questions about the number of hours participants expected to work and usually worked from home in a typical week (Appendix Figure 1). We categorized as having only onsite experience employed persons who reported that they usually worked no hours from home. We categorized as having hybrid (both onsite and remote) experience persons who stated that hours worked from home were usually fewer than total hours they expected to work. We categorized remaining persons as having only remote experience. 

We categorized daily work attendance based on whether persons scheduled to work did or did not work. We categorized persons as scheduled to work for a given day regardless of number of hours for which they were scheduled. Among persons scheduled to work, we categorized those who worked for any number of hours, even if not total hours scheduled, as having worked and remaining persons as having not worked (Appendix Figure 2). We categorized persons who reported work location for a given day as onsite only or hybrid as having worked onsite. 

We classified laboratory-confirmed influenza and SARS-CoV-2 viruses on the basis of positive results from PCR tests. We categorized persons with respiratory symptoms but negative PCR test results for influenza or SARS-CoV-2 as having other ARI. 

### Assembly of Participants

Among participants, 61% (12,941/21,133) completed the follow-up survey within 28 days of illness onset (Appendix Figure 3). Survey completion rates were 39% for Texas, 43% for Michigan, 60% for Washington, 75% for California, 75% for Pennsylvania, 79% for Wisconsin, and 89% for Tennessee. Among those who completed the follow-up survey, 69% (8,936/12,941) worked ≥20 h/wk before their illness. After excluding persons missing information on hours usually worked from home before illness or with indeterminate or missing laboratory results, we included 91% (8,132/8,936) in the analysis. 

### Statistical Analysis

We used χ^2^ testing to assess differences between frequencies of categorical variables and Wilcoxon rank-sum test to compare differences in spread and medians (*16*). We computed adjusted odds ratios (aOR) for each day by fitting multilevel logistic regression models to account for clustering of participants within study sites using PROC GLIMMIX in SAS version 9.4 (SAS Institute, https://www.sas.com). We ran 2 sets of regressions for employed persons who were scheduled to work. For the first set of regressions, the dependent variable was having worked at any location. For the second set of regressions, which examined work location to assess the worker’s potential to infect coworkers, the dependent variable was worked onsite. Because persons with remote-only experience before illness onset were unlikely to work onsite while ill, we excluded them from analyses pertaining to work location. 

We used a backward selection process using change in –2 log likelihood to assess model fit to determine retention of independent variables in the models and ultimately dropped age, sex, education, and number of children in the household. We then assessed interactions between remaining independent variables (Table 1–4; Appendix Tables 7, 8, 9). 

**Table 1:** Likelihood of working at any location among adults with COVID-19, influenza, or other acute respiratory illness who were scheduled to work, by work experience in a typical week before illness onset, United States, 2018–2022*

Period	Day before illness	Day 1 of illness	Day 2 of illness	Day 3 of illness
Prepandemic influenza seasons	n = 1,409	n = 2,596	n = 2,444	n = 2,373
Unadjusted analysis				
Work experience†				
Remote only	97.5 (39/40)	70.5 (43/61)	66.7 (40/60)‡	68.4 (39/57)‡
Hybrid	90.6 (222/245)	72.6 (329/453)	68.0 (297/437)	63.3 (274/433)
Onsite only	92.3 (1,037/1,124)	69.4 (1,445/2,082)	51.4 (1,000/1,947)	48.4 (912/1,883)
Adjusted analysis§				
Work experience				
Remote only	NA	1.02 (0.57–1.84)	1.90 (1.06–3.39)	2.13 (1.16–3.91)
Hybrid	NA	1.01 (0.78–1.30)	1.92 (1.50–2.46)	1.66 (1.30–2.12)
Onsite only	NA	Referent	Referent	Referent
COVID-19 pandemic period	n = 2,738	n = 3,178	n = 3,090	n = 3,040
Unadjusted analysis				
Work experience†				
Remote only	95.8 (498/520)‡	80.5 (495/615)‡	71.7 (451/629)‡	72.4 (449/620)‡
Hybrid	95.6 (540/565)	78.4 (514/656)	68.9 (451/655)	65.2 (416/638)
Onsite only	90.1 (1,490/1,653)	65.1 (1,242/1,907)	41.6 (752/1,806)	37.4 (666/1,782)
Adjusted analysis§				
Work experience				
Remote only	NA	2.03 (1.58–2.59)	3.37 (2.68–4.23)	3.78 (3.00–4.77)
Hybrid	NA	1.69 (1.34–2.13)	2.75 (2.22–3.42)	2.56 (2.06–3.19)
Onsite only	NA	Referent	Referent	Referent

*NA, not applicable. †Values are percentage (no. persons worked at any location/no. persons scheduled to work). ‡p<0.001 (comparison of 3 work experience categories for specified day and period). §Values are adjusted odds ratio (95% CI). Dependent variable for the multilevel logistic regression models is worked at any location on a specified day of illness (0 = did not work, 1 = worked). Independent variables are work experience in a typical week before illness onset (remote only, hybrid, onsite only), study period (0 = prepandemic influenza seasons, 1 = COVID-19 pandemic period). PCR test result (0 = other acute respiratory illness, 1 = influenza or COVID-19), race/ethnicity, general health before illness, current smoker, type of employment, healthcare personnel, hours worked in a typical week before illness onset, employees discouraged from coming to work with flu-like symptoms, and study site. We excluded persons with missing information for independent variables (303 for day 1, 314 for day 2, and 279 for day 3). p<0.001 for work experience study period interaction term for days 1–3 of illness.

## Results

During the prepandemic influenza seasons, 1,245 persons had confirmed influenza and 2,362 other ARI (Appendix Figure 4). During the COVID-19 pandemic period, 114 persons had influenza, 1,888 had COVID-19, and 2,523 had other ARI. Among persons in the study with any respiratory illness, 82.6% with influenza, 61.4% with COVID-19, and 49.6% with other ARI reported having fever. 

Among all participants, 14.0% (1,139) had only remote experience before illness onset, 18.5% (1,503) had hybrid experience, and 67.5% (5,490) had only onsite experience (Appendix Table 3). Hourly workers made up a significantly lower percentage of persons with remote-only (29.9%) or hybrid (21.8%) experience than onsite-only experience (66.6%) (p<0.001). Percentages of participants working in healthcare by location of work experience varied: 7.1% of remote-only, 15.5% of hybrid, and 25.4% of onsite-only personnel (p<0.001). Percentage of participants with at least a bachelor’s degree was significantly higher among persons with remote-only (71.3%) or hybrid (79.5%) experience than those with onsite-only experience (43.5%; p<0.001). Among 1,139 persons with remote-only experience during the study period, most (88.9%) were enrolled during the pandemic period. Among the 1,503 persons with hybrid experience, median hours worked from home in a typical week before illness onset was significantly higher during the pandemic period (16 h/wk) than during prepandemic influenza seasons (8 h/wk; p<0.001). 

Approximately three fourths of participants were scheduled to work on each of the first 3 days after illness onset (Appendix Table 4). Persons with previous remote-only or hybrid experience were significantly more likely than those with only onsite experience to work at any location on the second and third days of illness (Table 1). For example, on the third day of illness during the pandemic period, the percentage who worked at any location was 72.4% for persons with remote-only experience, 65.2% for persons with hybrid experience, and 37.4% for those with onsite-only experience (p<0.001). Among all persons who worked at any location on scheduled work days, median time worked was 8 (interquartile range 8–8) hours for the day before illness and 8 (interquartile range 6–8) hours for each of the first 3 days of illness (Appendix Table 5). Analysis of the location of work showed that participants were significantly more likely to work remotely on the day before illness onset through the first 3 days of illness during the pandemic period than prepandemic influenza seasons (Table 2). For example, on the third day of illness, 18.5% of persons worked remotely during the pandemic period, compared with 8.8% during the prepandemic influenza seasons. 

**Table 2:** Reported work location among adults with influenza, COVID-19, or other acute respiratory illness who were scheduled to work, United States, 2018–2022*

Period	No. (%)			
	Day before illness	Day 1 of illness	Day 2 of illness	Day 3 of illness
Prepandemic influenza seasons	n = 1,358	n = 2,515	n = 2,372	n = 2,304
Work location				
Onsite only	1,161 (85.5)†	1,464 (58.2)†	1,002 (42.2)†	920 (39.9)†
Hybrid	41 (3.0)	75 (3.0)	66 (2.8)	52 (2.3)
Remote only	46 (3.4)	215 (8.5)	217 (9.2)	202 (8.8)
Did not work	110 (8.1)	761 (30.3)	1,087 (45.8)	1,130 (49.0)
COVID-19 pandemic period	n = 2,188	n = 2,509	n = 2,418	n = 2,382
Work location				
Onsite only	1,676 (76.6)	1,239 (49.4)	644 (26.6)	561 (23.5)
Hybrid	73 (3.3)	83 (3.3)	42 (1.8)	43 (1.8)
Remote only	251 (11.5)	380 (15.1)	474 (19.6)	440 (18.5)
Did not work	188 (8.6)	807 (32.2)	1,258 (52.0)	1,338 (56.2)

*We excluded persons with only remote work experience before illness onset (560 for day before illness, 676 for day 1, 689 for day 2, and 677 for day 3) and those with missing work location (41 for day before illness, 74 for day 1, 55 for day 2, and 50 for day 3). †p<0.001 (comparison of prepandemic to pandemic period for specified day).

Participants with hybrid experience were less likely to work onsite than persons with onsite-only experience on the day before through the first 3 days of illness (Table 3); effect magnitude was more pronounced during the pandemic period than prepandemic influenza seasons. For example, for the third day of illness, hybrid versus onsite-only aOR was greater for the pandemic (aOR 0.38, 95% CI 0.29–0.49) than the prepandemic period (aOR 0.69, 95% CI 0.54–0.87; p<0.001 for the work experience–study period interaction term). Conversely, participants were less likely to work onsite during the pandemic period than prepandemic influenza seasons and effect magnitude was more pronounced among persons with hybrid than onsite-only experience. For example, for the third day of illness, pandemic versus prepandemic aOR was greater among persons with hybrid (0.32) than onsite-only (0.59) experience (Table 3). Persons with hybrid experience were more likely to work remotely during the pandemic period than they were during the prepandemic period (Appendix Table 6). In contrast, persons with onsite-only experience were more likely not to work on scheduled-to-work days during the pandemic than during the prepandemic period. Findings were similar even when we restricted data for the regression models to non–healthcare personnel or the sites that contributed data for all 4 study years (Appendix Tables 7, 8). Findings were also similar when we restricted the analysis to the sites with highest survey completion rates (Appendix Table 9). 

**Table 3:** Likelihood of working onsite among adults with influenza, COVID-19, or other acute respiratory illness who were scheduled to work, by work experience in a typical week before illness onset, United States, 2018–2022*

Category	Day before illness, n = 3,546	Day 1 of illness, n = 5,024	Day 2 of illness, n = 4,790	Day 3 of illness, n = 4,686
Prepandemic influenza seasons				
Work experience				
Hybrid	77.3 (187/242)†	52.2 (234/448)†	42.8 (186/435)	36.9 (158/428)‡
Onsite only	91.0 (1,015/1,116)	63.1 (1,305/2,067)	45.5 (882/1,937)	43.4 (814/1,876)
aOR (95% CI)¶	0.33 (0.22–0.48)#	0.62 (0.49–0.77)	0.90 (0.72–1.13)	0.69 (0.54–0.87)
COVID-19 pandemic period				
Work experience				
Hybrid	57.0 (317/556)†	33.2 (212/638)†	14.4 (92/640)†	14.3 (89/624)†
Onsite only	87.8 (1,432/1,632)	59.3 (1,110/1,871)	33.4 (594/1,778)	29.3 (515/1,758)
aOR (95% CI)¶	0.16 (0.13–0.21)	0.33 (0.27–0.41)	0.35 (0.27–0.46)	0.38 (0.29–0.49)
Hybrid				
Period				
COVID-19 pandemic	57.0 (317/556)†	33.2 (212/638)†	14.4 (92/640)†	14.3(89/624)†
Prepandemic influenza seasons	77.3 (187/242)	52.2 (234/448)	42.8 (186/435)	36.9 (158/428)
aOR (95% CI)¶	0.38 (0.27–0.55)	0.52 (0.40–0.68)	0.27 (0.20–0.37)	0.32 (0.23–0.45)
Onsite only				
Period				
COVID-19 pandemic	87.8 (1,432/1,632)§	59.3 (1,110/1,871)‡	33.4 (594/1,778)†	29.3 (515/1,758)†
Prepandemic influenza seasons	91.0 (1,015/1,116)	63.1 (1,305/2,067)	45.5 (882/1,937)	43.4 (814/1,876)
aOR (95% CI)¶	0.77 (0.59–1.01)	0.96 (0.83–1.11)	0.69 (0.60–0.80)	0.59 (0.51–0.69)

*Values are percentage (no. persons worked onsite/no. persons scheduled to work) except as indicated. Worked onsite represents onsite only or hybrid work location. We excluded persons with only remote work experience before illness onset (560 for day before illness, 676 for day 1, 689 for day 2, and 677 for day 3) and those with missing work location (41 for day before illness, 74 for day 1, 55 for day 2, and 50 for day 3). aOR, adjusted odds ratio. †p<0.001 (comparison of % working onsite for specified day). ‡p<0.05 (comparison of % working onsite for specified day). §p<0.01 (comparison of % working onsite for specified day). ¶Dependent variable in the multilevel logistic regression models is worked onsite on a specified day (0 = did not work or worked remotely only, 1 = worked onsite [onsite only or hybrid]). Independent variables are work experience in a typical week before illness onset (0 = onsite only, 1 = hybrid), study period (0 = Prepandemic influenza seasons, 1 = COVID-19 pandemic period), PCR test result (0 = Other acute respiratory illness, 1 = Influenza or COVID-19), race/ethnicity, general health before illness, current smoker, type of employment, healthcare personnel, hours worked in a typical week before illness, employees discouraged from coming to work with flu-like symptoms, and study site. We excluded persons with missing information for independent variables (173 for day before illness, 237 for day 1, 247 for day 2, and 216 for day 3) in addition to those mentioned above. p<0.01 for work experience study period interaction term for day before illness; p<0.001 for work experience study period interaction term for days 1–3 of illness. #For the October 2019–March 2020 prepandemic influenza season when all participating sites collected data on work status and location of work on the day before illness onset, aOR for the day before illness = 0.35 (95% CI 0.22–0.57).

We stratified the analysis by PCR test results, which showed that the proportion of employees who did not work while ill was greater for persons with influenza or COVID-19 than for persons with other ARI (Appendix Table 10). During prepandemic influenza seasons, 64.4% of persons with influenza and 40.3% for persons with other ARI did not work on the third day of illness (p<0.001). During the pandemic period, 66.7% of persons with COVID-19 and 48.3% of persons with other ARI did not work on the third day of illness (p<0.001). 

For the prepandemic influenza seasons, persons with influenza were significantly less likely than persons with other ARI to work onsite on the second (aOR 0.51, 95% CI 0.43–0.61) and third (aOR 0.39, 95% CI 0.32–0.47) days of illness (Table 4). For the pandemic period, participants with COVID-19 were also significantly less likely than persons with other ARI to work onsite on the second (aOR 0.59, 95% CI 0.49–0.73) or third (aOR 0.31, 95% CI 0.25–0.39) days of illness. Among persons with influenza, COVID-19, or other ARI, those with fever were less likely to work onsite than those with no fever (Appendix Table 11). 

**Table 4:** Likelihood of working onsite among adults who were scheduled to work, by PCR test result, United States, 2018–2022*

Category	Day before illness, n = 3,489	Day 1 of illness, n = 4,959	Day 2 of illness, n = 4,720	Day 3 of illness, n = 4,619
Prepandemic influenza seasons				
Influenza	88.8 (443/499)	59.3 (504/850)	34.1 (285/835)†	28.2 (236/837)†
Other ARI	88.4 (759/859)	62.2 (1,035/1,665)	50.9 (783/1,537)	50.2 (736/1,467)
aOR (95% CI)‡	1.01 (0.70–1.46)	0.92 (0.77–1.10)	0.51 (0.43–0.61)	0.39 (0.32–0.47)
COVID-19 pandemic period				
COVID-19§	78.7 (681/865)	51.2 (522/1,020)	22.3 (220/986)†	14.1 (137/974)†
Other ARI	81.4 (1,031/1,266)	53.9 (768/1,424)	33.0 (450/1,362)	33.7 (452/1,341)
aOR (95% CI)‡	0.80 (0.63–1.01)	0.92 (0.77–1.09)	0.59 (0.49–0.73)	0.31 (0.25–0.39)
Influenza or COVID-19				
COVID-19 pandemic period	78.7 (681/865)†	51.2 (522/1,020)†	22.3 (220/986)†	14.1 (137/974)†
Prepandemic influenza seasons	88.8 (443/499)	59.3 (504/850)	34.1 (285/835)	28.2 (236/837)
aOR (95% CI)‡	0.53 (0.37–0.75)	0.84 (0.69–1.03)	0.65 (0.52–0.81)	0.45 (0.35–0.58)
Other ARI				
COVID-19 pandemic period	81.4 (1,031/1,266)†	53.9 (768/1,424)†	33.0 (450/1,362)†	33.7 (452/1,341)†
Prepandemic influenza seasons	88.4 (759/859)	62.2 (1,035/1,665)	50.9 (783/1,537)	50.2 (736/1,467)
aOR (95% CI)‡	0.67 (0.51–0.89)	0.84 (0.72–0.99)	0.56 (0.47–0.66)	0.56 (0.48–0.67)

*Values are percentage (no. persons worked onsite/no. persons scheduled to work) except as indicated. Worked onsite represents onsite only or hybrid work location. We excluded persons with influenza during the COVID-19 pandemic period (57 for day before illness, 65 for day 1, 70 for day 2, and 67 for day 3), persons with only remote work experience before illness onset period (560 for day before illness, 676 for day 1, 689 for day 2, and 677 for day 3), persons with missing work location (41 for day before illness, 74 for day 1, 55 for day 2, and 50 for day 3). ARI, acute respiratory illness; aOR, adjusted odds ratio. †p<0.001 (comparison of % working onsite for specified day). ‡Dependent variable in the multilevel logistic regression models is worked onsite on a specified day (0 = did not work or worked remotely only, 1 = worked onsite [onsite only or hybrid]). Independent variables are work experience in a typical week before illness onset (0 = onsite only, 1 = hybrid), study period (0 = prepandemic influenza seasons, 1 = COVID-19 pandemic period), PCR test result (0 = other acute respiratory illness, 1 = influenza or COVID-19), race/ethnicity, general health before illness, current smoker, type of employment, healthcare personnel, hours worked in a typical week before illness, employees discouraged from coming to work with flu-like symptoms, and study site. We excluded persons with missing information for independent variables (170 for day before, 237 for day 1, 247 for day 2, and 216 for day 3) in addition to those mentioned above. §Among persons with COVID-19, healthcare personnel were significantly more likely to work onsite than nonhealthcare personnel on the day before illness (85.9% vs 76.7%, p<0.01) and the first day of illness (58.4% vs.49.2%, p<0.05), but not on the second (22.0% vs. 22.3%) and third (11.7% vs. 14.7%) days of illness.

Among persons with COVID-19, substantial percentages worked onsite while ill: 51.2% on day 1, 22.3% on day 2, and 14.1% on day 3 (Table 4). COVID-19–positive PCR test results were available for 1.3% (12/940) by the first day of COVID-19 illness, 10.7% (97/910) by the second day, and 23.5% (211/899) by the third day (Table 5). Persons for whom a positive COVID-19 PCR test result was available by the second day of illness were significantly less likely to work onsite on that day than those whose positive PCR result was available on the third day or later (5.2% vs. 25.0%; p<0.001) (Table 5). Persons for whom a positive PCR test result was available by the third day of illness were significantly less likely to work onsite on that day than those whose positive PCR result was available later than the third day of illness (4.7% vs. 17.2%; p<0.001). Among persons for whom positive PCR test results were available after the second or third day of illness, the percentage who worked onsite was slightly higher when we excluded persons with COVID-19–positive at-home test results by the second or third day of illness (Appendix Table 12). 

**Table 5:** Likelihood of working onsite among adults with COVID-19 illness who were scheduled to work, by day when COVID-19–positive PCR test result was available, United States, October 2020–June 2022*

Characteristic	Value
Scheduled to work on day 1 of COVID-19 illness†	
COVID-19–positive PCR result available on day 1 of illness	50.0 (6/12)
COVID-19–positive PCR result available after day 1 of illness	52.1 (483/928)
Scheduled to work on day 2 of COVID-19 illness‡	
COVID-19–positive PCR result available on day 1 or 2 of illness	5.2 (5/97)§
COVID-19–positive PCR result available after day 2 of illness	25.0 (203/813)
Scheduled to work on day 3 of COVID-19 illness¶	
COVID-19–positive PCR result available on day 1, 2, or 3 of illness	4.7 (10/211)§
COVID-19–positive PCR result available after day 3 of illness	17.2 (118/688)

*Day of illness when COVID-19 positive result was available was computed by comparing the date of illness onset with the date that COVID-19–positive PCR test result was available. Values represent % worked onsite (no. worked onsite/no. scheduled to work). Worked onsite represents onsite only or hybrid work location. Analysis was restricted to persons with COVID-19 as shown in Table 4. †Unknown when COVID-19 positive PCR result was available = 80 persons. ‡Unknown when COVID-19 positive PCR result was available = 76 persons. §p<0.001. ¶Unknown when COVID-19 positive PCR result was available = 75 persons.

## Discussion

During both prepandemic and pandemic periods, adults with remote-only or hybrid experience were more likely to work within the first 3 days of illness compared with those with onsite-only experience. It is notable, however, that persons with hybrid experience were significantly less likely to work onsite on the day before illness through the first 3 days of illness than those with only onsite experience. The effect magnitude of hybrid compared with onsite-only experience on working onsite while ill was more pronounced for the pandemic period than for the prepandemic period. Persons with influenza or COVID-19 were significantly less likely to work onsite on the second and third days of illness than were persons with other ARI. For persons for whom a positive COVID-19 PCR test result was available by the second or third day of illness, few reported working onsite. 

Persons with previous remote-only or hybrid experience were significantly more likely to work at any location while ill than those with only onsite experience, enabling a greater level of continuity of work while ill. Greater likelihood of working at any location among persons with hybrid experience than those with only onsite experience has been reported in studies conducted during the 2017–2018 influenza season and during the early part of the COVID-19 pandemic (March–November 2020) (*10*,*17*). Remote-only or hybrid experience before illness can enable persons to work remotely if they are well enough, instead of taking sick days. 

It is possible that persons without experience working from home were more likely to work in occupations in which remote-only or hybrid work is less feasible and, therefore, workers are less likely to have the option or incentive to work remotely. Those workers might include persons with jobs in hospitality and leisure, transportation, utilities, construction, production, and agriculture (*18*,*19*). 

Employers were required to provide paid sick leave to workers with COVID-19 during the pandemic (*20*). It is unlikely that persons with only onsite experience worked less than persons with hybrid experience after testing SARS-CoV-2–positive because they received paid sick leave. This pattern of persons with only onsite experience working less than persons with hybrid experience was also observed during the pandemic influenza seasons. 

Persons with previous hybrid experience were less likely to work onsite the day before illness onset through the first 3 days of illness than persons with only onsite experience, thus reducing the likelihood of workplace exposures to respiratory viruses. A study conducted during the 2017–18 influenza season concurred with that finding, but the study did not examine the likelihood of working onsite on the day before illness (*17*). A study conducted during the early part of the COVID-19 pandemic found that persons with hybrid experience were less likely to work onsite while ill than were persons with only onsite experience (*10*), an effect more pronounced during the pandemic than the prepandemic period. That difference may have been because of the greater prevalence of telework regardless of illness status during the pandemic (*3*,*4*). During the pandemic period, intense public health messaging to stay home when ill, employer policies discouraging or prohibiting employees with ARI symptoms from working onsite, and provision of flexible paid leave for persons with COVID-19 illness may have contributed to the greater effect (*5*,*20*). 

Persons with laboratory-confirmed influenza or COVID-19 were significantly less likely than persons with other ARI to work onsite on the second and third days of illness. Previous studies have reported similar findings but did not assess the likelihood of working onsite on each of the first 3 days of illness (*10*,*17*). Those findings might be attributable to more severe manifestations of illness in persons with influenza or COVID-19 (*15*). The finding that the likelihood of working onsite was similar in persons with influenza or COVID-19 compared with persons with other ARI on the first day of illness, as well as the greater likelihood of working onsite on the first day of illness compared with the second or third day of illness, might have been because illness had begun when participants were already at work. For persons ill with COVID-19, having positive PCR test results by the second or third day of illness might have reduced the likelihood of working onsite for several reasons, including being concerned for coworkers, being advised to isolate by case investigators, having employers discourage or prohibit persons with COVID-19 from entering the worksite, and having employers provide flexible sick leave. However, COVID-19–positive PCR test results were available for only a small proportion of persons within the first 3 days of illness because of the lag between illness onset and seeking medical care. At-home rapid COVID-19 tests may enable early testing for persons with symptoms of ARI. Use of at-home tests among persons with COVID-19–like illness in the United States increased from 6% during August 23–December 11, 2021, to 20% during December 19, 2021–March 12, 2022 (*21*). 

Strengths of our study were that we included data from ≈8,000 persons over a 4-year study period that encompassed both prepandemic and pandemic periods. We obtained respiratory specimens that enabled laboratory confirmation of influenza and SARS-CoV-2. Also, we assessed work attendance within the presymptomatic phase, when persons can be infectious, and the first 3 days of illness, when infectiousness is greatest. One limitation of the study was that 39% of participants did not complete the follow-up survey. However, findings were similar when we restricted the analysis to the sites with the highest survey completion rates. Second, we assessed the proportion of employees who worked at any location within the first 3 days of illness as an indicator of maintenance of workflow. We did not assess how illness may have diminished productivity among persons working while ill versus those working while well. Third, we assessed work attendance only among persons with medically attended ARIs. Findings may not be generalizable to persons who were asymptomatic or who did not seek medical care. 

Future research should ascertain productivity in persons who work while ill with influenza or COVID-19. In addition, an assessment of the likelihood of working onsite among persons with ARI who do not seek medical care is needed. Research is also needed on how type of occupation and other workplace policies affect work attendance of sick employees. 

In conclusion, working-age adults continue to be at risk for severe COVID-19 (*22*). Our study findings show that hybrid work experience before illness onset might give workers the opportunity to continue working but also reduce time worked onsite early in illness, when infectiousness is high. When feasible for a given occupation, employers should consider hybrid and remote work policies that might reduce likelihood of workplace exposures to influenza and SARS-CoV-2 viruses. Such work policies could minimize interaction with infectious persons in workplaces during both the presymptomatic and symptomatic phases of illness and help reduce spread of respiratory viruses. 

Appendix Additional information about study of work attendance with acute respiratory illness before and during the COVID-19 pandemic, United States, 2018–2022.
