# Supplementary material for: Work Attendance with Acute Respiratory Illness Before and During COVID-19 Pandemic, United States, 2018–2022
Source: Emerg Infect Dis. 2023 Dec;29(12):2442–50. doi: 10.3201/eid2912.231070 (PMC10683820; doi:10.3201/eid2912.231070)
Supplement: Appendix — Additional information about study of work attendance with acute respiratory illness before and during the COVID-19 pandemic, United States, 2018–2022. [file 23-1070-Techapp-s1.pdf]

Article DOI: https://doi.org/10.3201/eid2912.231070

EID cannot ensure accessibility for supplementary materials supplied by authors. Readers who have difficulty accessing supplementary content should contact the authors for assistance.

Work Attendance with Acute Respiratory Illness Before and During the COVID-19 Pandemic, United States, 2018–2022

Appendix

Appendix Table 1. Follow-up survey questions, 2018–2022*

Question	Values
Have you fully or mostly recovered from this illness?	Yes: Date: __-__-____ (mm/dd/yyyy)<br>I have not fully or mostly recovered from this illness<br>Don’t know<br>Refused
Are you currently employed (work for pay or profit)?	I work for an employer<br>I am self-employed or own my own business ➔ [Survey is complete]<br>No ➔ [Survey is complete]<br>Refused ➔ [Survey is complete]
How many hours are you expected to work in a typical 7-day week?†<br>(If it varies, estimate the average)	Number of hours
Of those expected hours, how many hours in a week do you usually work from home (telework, telecommute, or remote work)?‡<br>(Enter “0” if none)	Number of hours
Are you salaried or are you paid hourly? [“Salaried” means you’re paid the same amount each week or month no matter how many hours you work. “Hourly” means that you’re paid a different amount each week or month depending on how many hours you work.]	Salaried<br>Paid hourly<br>Other such as commission only<br>Don’t know<br>Refused
Do you work in a healthcare setting with direct patient contact?§	Yes<br>No<br>Don’t know<br>Refused
Please think about the first three days of your illness. The first day of illness being the day your symptoms started [DAY/DATE OF ONSET] and the third day of illness being [DATE OF ONSET + 2].	
On the first day of illness [DATE OF ONSET]:	
How many hours were you scheduled to work?	Number of hours
How many hours did you work?	Number of hours
Where did you work?	At work<br>Remotely<br>Both at work and remotely<br>Don’t know<br>Refused
On the second day of illness [DATE OF ONSET+1]:	
How many hours were you scheduled to work?	Number of hours
How many hours did you work?	Number of hours
Where did you work?	At work<br>Remotely<br>Both at work and remotely<br>Don’t know<br>Refused

Question	Values
On the third day of illness [DATE OF ONSET+2]:	
How many hours were you scheduled to work?	Number of hours
How many hours did you work?	Number of hours
Where did you work?	At work<br>Remotely<br>Both at work and remotely<br>Don't know<br>Refused
On the day before your illness began [DATE OF ONSET-1]:¶	
How many hours were you scheduled to work?	Number of hours
How many hours did you work?	Number of hours
Where did you work?	At work<br>Remotely<br>Both at work and remotely<br>Don't know<br>Refused
Please select your level of agreement with the following statement about your place of work:<br>- Employees are discouraged from coming to work when they have flu-like symptoms	Strongly agree<br>Agree<br>Neither agree nor disagree<br>Disagree<br>Strongly disagree

*If a person worked multiple jobs, the person was asked to think about the job that they considered as their primary job when answering the questions.
†For the COVID-19 pandemic period, the wording was "During the month before illness, how many hours were you expected to work in a week?".
‡For the COVID-19 pandemic period, the wording was: "Of those expected hours, how many hours in a week did you usually work from home (telework, telecommute, or remote work)?
§For the COVID-19 pandemic period, the question was asked at enrollment: "Did you work in a healthcare setting and have close contact with patients during the 14 days before your illness began? Close contact means being within 6 feet of a patient."
¶This was an optional question for the period November 2018–May 2019.

Appendix Table 2. Data collection by study sites, 2018–2022*

	Nov 2018–May 2019		Oct 2019–Mar 2020		Oct 2020–Sep 2021		Oct 2021–Jun 2022	
Site	D0	D1, D2, D3	D0	D1, D2, D3	D0	D1, D2, D3	D0	D1, D2, D3
MI		✓	✓	✓	✓	✓	✓	✓
PA	✓	✓	✓	✓	✓	✓	✓	✓
TX		✓	✓	✓	✓	✓	✓	✓
WA†		✓	✓	✓	✓	✓	✓	✓
WI†		✓	✓	✓	✓	✓	✓	✓
CA							✓	✓
TN							✓	✓

*Check mark indicates that a site collected data for the specified period and day of illness. D0, day before illness; D1, first day of illness; D2, second day of illness; D3, third day of illness; MI, Michigan; PA, Pennsylvania; TX, Texas; WA, Washington; WI, Wisconsin; CA, California; TN, Tennessee.
†For persons with only remote work experience before illness onset, the Washington and Wisconsin sites did not collect data on work status (hours scheduled to work, hours worked) and location of work for the period November 2018 to September 2021.

Appendix Table 3. Characteristics of adults with COVID-19, influenza, or other acute respiratory illness, United States, 2018–2022*

	Work experience in a typical week before illness onset		
Characteristic	Remote only (n = 1,139)	Hybrid (n = 1,503)	Onsite only (n = 5,490)
Study period§			
Prepandemic influenza seasons	126 (11.1)	628 (41.8)	2,853 (52.0)
COVID-19 pandemic period	1,013 (88.9)	875 (58.2)	2,637 (48.0)
Median hours worked in a typical week before illness (IQR)§	40 (40, 40)	40 (40, 45)	40 (40, 40)
Median hours usually worked from home in a typical week before illness (IQR)			
Prepandemic influenza seasons	40 (40, 45)†	8 (5, 15)§	0 (0, 0)
COVID-19 pandemic period	40 (40, 40)	16 (8, 25)	0 (0, 0)
Type of employment§			
Hourly	281 (29.9)	325 (21.8)	3,618 (66.6)
Salaried or other	658 (70.1)	1,166 (78.2)	1,816 (33.4)
Healthcare personnel§			
Yes	78 (7.1)	231 (15.5)	1,379 (25.4)
No	1,017 (92.9)	1,264 (84.5)	4,055 (74.6)
Employees discouraged from coming to work with flu-like symptoms§			

Characteristic	Work experience in a typical week before illness onset		
	Remote only (n = 1,139)	Hybrid (n = 1,503)	Onsite only (n = 5,490)
Agree	805 (88.6)	1,293 (86.8)	4,175 (76.7)
Not agree	104 (11.4)	196 (13.2)	1,266 (23.3)
Median age, y (IQR)§	40 (33, 51)	40 (33, 51)	39 (30, 51)
Sex‡			
Female	781 (68.8)	939 (62.5)	3,651 (66.5)
Male	354 (31.2)	564 (37.5)	1,835 (33.5)
Race/ethnicity‡			
White, non-Hispanic	846 (74.8)	1,142 (76.5)	4,222 (77.4)
Black, non-Hispanic	63 (5.6)	68 (4.5)	335 (6.1)
Other, non-Hispanic	122 (10.8)	152 (10.2)	422 (7.7)
Hispanic, any race	100 (8.8)	131 (8.8)	479 (8.8)
Education§			
High school or less	90 (7.9)	50 (3.3)	1,073 (19.6)
Some college	236 (20.8)	258 (17.2)	2,021 (36.9)
Bachelor’s degree	471 (41.4)	595 (39.7)	1,503 (27.4)
Advanced degree	340 (29.9)	597 (39.8)	883 (16.1)
General health before illness‡			
Excellent	203 (18.4)	316 (21.4)	991 (18.3)
Very good	473 (42.9)	652 (44.1)	2,244 (41.5)
Good	347 (31.5)	418 (28.3)	1,743 (32.2)
Fair/poor	79 (7.2)	91 (6.2)	434 (8.0)
Current smoker§			
Yes	57 (5.1)	99 (6.7)	647 (11.9)
No	1,059 (94.9)	1,378 (93.3)	4,777 (88.1)
Children <12 y of age in household†			
0	745 (65.7)	1,002 (67.0)	3,812 (69.6)
1	199 (17.6)	252 (16.8)	810 (14.8)
≥2	190 (16.7)	242 (16.2)	856 (15.6)
Fully or mostly recovered from illness at follow-up			
Yes	834 (74.9)	1,079 (73.1)	3,879 (72.1)
No	279 (25.1)	398 (26.9)	1,499 (27.9)
Among persons fully or mostly recovered from illness, median duration of illness, days (IQR)‡	9 (7, 12)	9 (7,12)	9 (7, 11)
Median days from illness onset to enrollment (IQR)§	4 (2, 7)	3 (2, 6)	3 (2, 5)
Median days from illness onset to follow-up (IQR)§	13 (10, 17)	12 (10, 15)	12 (10, 14)
Site§			
Michigan	91 (8.0)	169 (11.2)	405 (7.4)
Pennsylvania	323 (28.4)	410 (27.3)	1,352 (24.6)
Texas	59 (5.2)	91 (6.1)	604 (11.0)
Washington	238 (20.9)	346 (23.0)	1,143 (20.8)
Wisconsin	172 (15.1)	182 (12.1)	1,354 (24.7)
California	136 (11.9)	169 (11.2)	299 (5.4)
Tennessee	120 (10.5)	136 (9.1)	333 (6.1)

*Values are no. (column %) except as indicated. Numbers may not sum to n because of missing data. IQR, interquartile range.
†p<0.05.
‡p<0.01.
§p<0.001.

Appendix Table 4. Proportion of persons with COVID-19, influenza, or other acute respiratory illness who were scheduled to work, United States, 2018–2022*

Work experience in a typical week before illness onset	Day before illness	Day 1 of illness	Day 2 of illness	Day 3 of illness
	Scheduled to work, %†			
Remote only	64.6 (560/867)	77.1 (676/877)	77.5‡ (689/889)	76.0‡ (677/891)
Hybrid	66.3 (810/1,221)	76.3 (1,109/1,454)	74.5 (1,092/1,465)	73.5 (1,071/1,458)
Onsite only	66.0 (2,777/4,210)	75.4 (3,989/5,290)	70.5 (3,753/5,326)	69.2 (3,665/5,300)
TOTAL	65.9 (4147/6,298)	75.8 (5,774/7,621)	72.1 (5,534/7,680)	70.8 (5,413/7,649)

*We excluded persons if data on hours scheduled to work or hours worked were missing: 1,834 on the day before illness, 511 on day 1, 452 on day 2, and 483 on day 3. Among the 1,834 persons who were excluded on the day before illness, 1,423 were excluded because the Michigan, Texas, Washington, and Wisconsin sites did not collect data on work status (hours scheduled to work, hours worked) for the day before illness during November 2018–May 2019.
†Numerator represents no. scheduled to work and denominator represents sum of no. scheduled to work and no. not scheduled to work. Among persons who were scheduled to work, the median (IQR) hours scheduled to work was 8 (8, 8) for the day before illness and for each day of illness.
‡p<0.001 (comparison of the three work experience categories for specified day).

Appendix Table 5. Hours worked among adults with COVID-19, influenza, or other acute respiratory illness who worked at any location, by work experience in a typical week before illness onset, United States, 2018–2022

Work experience	Day before illness	Day 1 of illness	Day 2 of illness	Day 3 of illness
Prepandemic influenza seasons	(n = 1,298)	(n = 1,817)	(n = 1,337)	(n = 1,225)
	Median hours worked (interquartile range)			
Remote only	8 (8, 8)	8 (6, 8)*	8 (4.5, 8)†	8 (6, 8)*
Hybrid	8 (8, 8)	8 (6, 8)	8 (5, 8)	8 (5, 8)
Onsite only	8 (8, 8)	8 (7, 8)	8 (7, 8)	8 (7, 8)
ALL ADULTS	8 (8, 8)	8 (6.5, 8)	8 (6, 8)	8 (6, 8)
COVID-19 pandemic period	(n = 2,528)	(n = 2,251)	(n = 1,654)	(n = 1,531)
	Median hours worked (interquartile range)			
Remote only	8 (8, 8)†	8 (6, 8)†	8 (6, 8)†	8 (6, 8)*
Hybrid	8 (8, 8)	8 (5, 8)	8 (4, 8)	8 (5, 8)
Onsite only	8 (8, 9)	8 (7, 8)	8 (6, 8)	8 (6, 8)
ALL ADULTS	8 (8, 8)	8 (6, 8)	8 (6, 8)	8 (6, 8)

*p<0.01 (comparison of the three work experience categories for specified day and period).
†p<0.001 (comparison of the three work experience categories for specified day and period).

Appendix Table 6. Reported work location among adults with influenza, COVID-19, or other acute respiratory illness who were scheduled to work, by work experience in a typical week before illness onset, United States, 2018-2022*

Location of work	Day before illness	Day 1 of illness	Day 2 of illness	Day 3 of illness
Hybrid experience				
COVID-19 pandemic period	(n = 556)	(n = 638)	(n = 640)	(n = 624)
Onsite only	255 (45.9)‡	155 (24.3)‡	66 (10.3)‡	59 (9.5)‡
Hybrid	62 (11.1)	57 (8.9)	26 (4.1)	30 (4.8)
Remote only	214 (38.5)	284 (44.5)	344 (53.8)	313 (50.2)
Did not work	25 (4.5)	142 (22.3)	204 (31.9)	222 (35.6)
Prepandemic influenza seasons	(n = 242)	(n = 448)	(n = 435)	(n = 428)
Onsite only	148 (61.2)	180 (40.2)	133 (30.6)	116 (27.1)
Hybrid	39 (16.1)	54 (12.0)	53 (12.2)	42 (9.8)
Remote only	32 (13.2)	90 (20.1)	109 (25.1)	111 (25.9)
Did not work	23 (9.5)	124 (27.7)	140 (32.2)	159 (37.2)
Onsite only experience				
COVID-19 pandemic period	(n = 1,632)	(n = 1,871)	(n = 1,778)	(n = 1,758)
Onsite only	1,421 (87.1)†	1,084 (57.9)†	578 (32.5)‡	502 (28.6)‡
Hybrid	11 (0.7)	26 (1.4)	16 (0.9)	13 (0.7)
Remote only	37 (2.3)	96 (5.1)	130 (7.3)	127 (7.2)
Did not work	163 (10.0)	665 (35.5)	1,054 (59.3)	1,116 (63.5)
Prepandemic influenza seasons	(n = 1,116)	(n = 2,067)	(n = 1,937)	(n = 1,876)
Onsite only	1,013 (90.8)	1,284 (62.1)	869 (44.9)	804 (42.9)
Hybrid	2 (0.2)	21 (1.0)	13 (0.7)	10 (0.5)
Remote only	14 (1.3)	125 (6.1)	108 (5.6)	91 (4.9)
Did not work	87 (7.8)	637 (30.8)	947 (48.9)	971 (51.8)

*We excluded persons with only remote work experience before illness onset (560, 676, 689, and 677 for day before, day 1, day 2, and day 3, respectively) and those with missing work location (41, 74, 55, and 50 for day before, day 1, day 2, and day 3, respectively).
†p<0.01 (comparison of work location for pandemic vs. prepandemic period for specified day).
‡p<0.001 (comparison of work location for pandemic vs. prepandemic period for specified day).

Appendix Table 7. Likelihood of working onsite among nonhealthcare personnel with COVID-19, influenza, or other acute respiratory illness who were scheduled to work, by work experience in a typical week before illness onset, United States, 2018–2022*

Work experience and period	Day before illness (n = 2,681)	Day 1 of illness (n = 3,849)	Day 2 of illness (n = 3707)	Day 3 of illness (n = 3,647)
Prepandemic influenza seasons				
Hybrid	76.3 (161/211)‡	52.3 (208/398)‡	43.3 (171/395)	37.4 (147/393)†
Onsite only	90.5 (770/851)	63.4 (999/1,577)	45.1 (672/1,491)	42.9 (628/1,464)
aOR (95% CI)§	0.34 (0.22–0.51)	0.60 (0.47–0.76)	0.92 (0.72–1.18)	0.73 (0.57–0.94)
COVID-19 pandemic period				
Hybrid	54.2 (237/437)‡	32.8 (167/509)‡	13.6 (68/501)‡	14.1 (69/491)‡
Onsite only	87.7 (1,036/1,182)	59.6 (814/1,365)	34.3 (453/1,320)	30.0 (389/1,299)
aOR (95% CI)§	0.15 (0.11–0.20)	0.31 (0.25–0.40)	0.31 (0.23–0.42)	0.35 (0.26–0.47)
Hybrid				
COVID-19 pandemic period	54.2 (237/437)‡	32.8 (167/509)‡	13.6 (68/501)‡	14.1 (69/491)‡
Prepandemic influenza seasons	76.3 (161/211)	52.3 (208/398)	43.3 (171/395)	37.4 (147/393)
aOR (95% CI)§	0.38 (0.26–0.55)	0.51 (0.38–0.68)	0.25 (0.18–0.36)	0.30 (0.21–0.43)
Onsite only				
COVID-19 pandemic period	87.7 (1,036/1,182)†	59.6 (814/1,365)†	34.3 (453/1,320)‡	30.0 (389/1,299)‡
Prepandemic influenza seasons	90.5 (770/851)	63.4 (999/1,577)	45.1 (672/1,491)	42.9 (628/1,464)
aOR (95% CI)§	0.85 (0.63–1.15)	0.98 (0.83–1.16)	0.74 (0.63–0.88)	0.63 (0.53–0.75)

*Values represent % worked onsite (no. worked onsite / no. scheduled to work) except as indicated. Worked onsite represents onsite only or hybrid work location. We excluded healthcare personnel, persons with only remote work experience before illness onset, and those with missing work location. aOR, adjusted odds ratio; CI, confidence interval.
†p<0.05 (comparison of % worked onsite for specified day).
‡p<0.001 (comparison of % worked onsite for specified day).
§Dependent variable in the multi-level logistic regression models is worked onsite during a specified day (0 = Did not work or worked remotely only, 1 = Worked onsite [onsite only or hybrid]). Independent variables are work experience in a typical week before illness onset (0 = Onsite only, 1 = Hybrid), study period (0 = Prepandemic influenza seasons, 1 = COVID-19 pandemic period), PCR test result (0 = Other acute respiratory illness, 1 = Influenza or COVID-19), race/ethnicity, general health before illness, current smoker, type of employment, healthcare personnel, hours worked in a typical week before illness, employees discouraged from coming to work with flu-like symptoms, and study site. We excluded persons with missing information for independent variables (92, 142, 147, and 130 for day before, day 1, day 2, and day 3, respectively) in addition to those mentioned above. p<0.001 for work experience study period interaction term for day before, day 1, day 2, and day 3 of illness.

Appendix Table 8. Likelihood of working onsite among adults with COVID-19, influenza, or other acute respiratory illness who were scheduled to work for the five sites that contributed data for all four years, by work experience in a typical week before illness onset, United States, 2018–2022*

Work experience and period	Day before illness (n = 2,955)	Day 1 of illness (n = 4,358)	Day 2 of illness (n = 4,130)	Day 3 of illness (n = 4,034)
Prepandemic influenza seasons				
Hybrid	77.3 (187/242)§	52.2 (234/448)§	42.8 (186/435)	36.9 (158/428)†
Onsite only	91.0 (1,015/1,116)	63.1 (1,305/2,067)	45.5 (882/1,937)	43.4 (814/1,876)
aOR (95% CI)¶	0.32 (0.21–0.47)	0.60 (0.48–0.75)	0.90 (0.71–1.13)	0.70 (0.55–0.89)
COVID-19 pandemic period				
Hybrid	58.1 (202/348)§	35.0 (142/406)§	14.2 (57/402)§	15.3 (60/393)§
Onsite only	87.6 (1,094/1,249)	60.5 (870/1,437)	35.1 (476/1,356)	29.9 (400/1,337)
aOR (95% CI)¶	0.17 (0.13–0.23)	0.32 (0.25–0.42)	0.32 (0.24–0.45)	0.41 (0.30–0.57)
Hybrid				
COVID-19 pandemic period	58.1 (202/348)§	35.0 (142/406)§	14.2 (57/402)§	15.3 (60/393)§
Prepandemic influenza seasons	77.3 (187/242)	52.2 (234/448)	42.8 (186/435)	36.9 (158/428)
aOR (95% CI)¶	0.40 (0.27–0.58)	0.52 (0.39–0.70)	0.25 (0.18–0.36)	0.35 (0.24–0.49)
Onsite only				
COVID-19 pandemic period	87.6 (1,094/1,249)‡	60.5 (870/1,437)	35.1 (476/1,356)§	29.9 (400/1,337)§
Prepandemic influenza seasons	91.0 (1,015/1,116)	63.1 (1,305/2,067)	45.5 (882/1,937)	43.4 (814/1,876)
aOR (95% CI)¶	0.74 (0.55–0.98)	0.96 (0.83–1.11)	0.71 (0.61–0.82)	0.59 (0.50–0.69)

*Values represent % worked onsite (no. worked onsite / no. scheduled to work) except as indicated. Worked onsite represents onsite only or hybrid work location. Michigan, Pennsylvania, Texas, Washington, and Wisconsin sites were included (California and Tennessee sites were excluded). We excluded persons with only remote work experience before illness onset and those with missing work location. aOR, adjusted odds ratio; CI, confidence interval.
†p<0.05 (comparison of % worked onsite for specified day).
‡p<0.01 (comparison of % worked onsite for specified day).
§p<0.001 (comparison of % worked onsite for specified day).
¶Dependent variable in the multi-level logistic regression models is worked onsite during a specified day (0 = Did not work or worked remotely only, 1 = Worked onsite [onsite only or hybrid]). Independent variables are listed in Appendix Table 7 footnote. We excluded persons with missing information for independent variables (131, 198, 205, and 176 for day before, day 1, day 2, and day 3, respectively) in addition to those mentioned above. p<0.01 for work experience study period interaction term for day before illness and third day of illness; p<0.001 for work experience study period interaction term for the first and second days of illness.

Appendix Table 9. Likelihood of working onsite among adults with COVID-19, influenza, or other acute respiratory illness who were scheduled to work for the five sites with the highest survey completion rates, by work experience in a typical week before illness onset, United States, 2018–2022*

Work experience and period	Day before illness (n = 3,057)	Day 1 of illness (n = 4,161)	Day 2 of illness (n = 3,965)	Day 3 of illness (n = 3,876)
Prepandemic influenza seasons				
Hybrid	76.1 (156/205)§	53.4 (179/335)§	43.0 (139/323)	37.3 (119/319)‡
Onsite only	90.8 (852/938)	67.5 (1,107/1,639)	48.5 (746/1,538)	45.5 (671/1,476)
aOR (95% CI)¶	0.30 (0.20–0.45)	0.57 (0.44–0.73)	0.84 (0.65–1.10)	0.67 (0.51–0.88)
COVID-19 pandemic period				
Hybrid	56.2 (284/505)§	33.5 (192/574)§	14.1 (81/574)§	13.6 (76/559)§
Onsite only	87.8 (1,237/1,409)	59.4 (958/1,613)	33.9 (519/1,530)	29.9 (455/1,522)
aOR (95% CI)¶	0.15 (0.12–0.20)	0.36 (0.29–0.45)	0.35 (0.26–0.46)	0.35 (0.26–0.47)
Hybrid				
COVID-19 pandemic period	56.2 (284/505)§	33.5 (192/574)§	14.1 (81/574)§	13.6 (76/559)§
Prepandemic influenza seasons	76.1 (156/205)	53.4 (179/335)	43.0 (139/323)	37.3 (119/319)
aOR (95% CI)¶	0.41 (0.28–0.60)	0.50 (0.37–0.67)	0.26 (0.18–0.37)	0.28 (0.20–0.41)
Onsite only				
COVID-19 pandemic period	87.8 (1,237/1,409)†	59.4 (958/1,613)§	33.9 (519/1,530)§	29.9 (455/1,522)§
Prepandemic influenza seasons	90.8 (852/938)	67.5 (1,107/1,639)	48.5 (746/1,538)	45.5 (671/1,476)
aOR (95% CI)¶	0.79 (0.59–1.06)	0.78 (0.67–0.92)	0.62 (0.53–0.74)	0.54 (0.46–0.64)

*Values represent % worked onsite (no. worked onsite / no. scheduled to work) except as indicated. Worked onsite represents onsite only or hybrid work location. California, Pennsylvania, Tennessee, Washington, and Wisconsin sites were included (Michigan and Texas sites were excluded). We excluded persons with only remote workexperience before illness onset and those with missing work location. aOR, adjusted odds ratio; CI, confidence interval.
†p<0.05 (comparison of % worked onsite for specified day).
‡p<0.01 (comparison of % worked onsite for specified day).
§p<0.001 (comparison of % worked onsite for specified day).
¶Dependent variable in the multi-level logistic regression models is worked onsite during a specified day (0 = Did not work or worked remotely only, 1 = Worked onsite [onsite only or hybrid]). Independent variables are listed in Appendix Table 7 footnote. We excluded persons with missing information for independent variables (159, 209, 216, and 190 for day before, day 1, day 2, and day 3, respectively) in addition to those mentioned above. p<0.01 for work experience study period interaction term for the day before illness and the first day of illness; p<0.001 for work experience study period interaction term for the second and third days of illness.

Appendix Table 10. Reported work location among adults with influenza, COVID-19, or other acute respiratory illness (ARI) who were scheduled to work, by PCR test result, United States, 2018-2022*

Location of work	Day before illness	Day 1 of illness	Day 2 of illness	Day 3 of illness
	No. (column %)			
Prepandemic influenza seasons				
Influenza	(n = 499)	(n = 850)	(n = 835)	(n = 837)
Onsite only	430 (86.2)	481 (56.6)†	272 (32.6)‡	227 (27.1)‡
Hybrid	13 (2.6)	23 (2.7)	13 (1.5)	9 (1.1)
Remote only	13 (2.6)	59 (6.9)	69 (8.3)	62 (7.4)
Did not work	43 (8.6)	287 (33.8)	481 (57.6)	539 (64.4)
Other ARI	(n = 859)	(n = 1,665)	(n = 1,537)	(n = 1,467)
Onsite only	731 (85.1)	983 (59.0)	730 (47.5)	693 (47.3)
Hybrid	28 (3.3)	52 (3.1)	53 (3.5)	43 (2.9)
Remote only	33 (3.8)	156 (9.4)	148 (9.6)	140 (9.5)
Did not work	67 (7.8)	474 (28.5)	606 (39.4)	591 (40.3)
COVID-19 pandemic period				
COVID-19	(n = 865)	(n = 1,020)	(n = 986)	(n = 974)
Onsite only	656 (75.8)†	488 (47.9)	209 (21.2)‡	124 (12.8)‡
Hybrid	25 (2.9)	34 (3.3)	11 (1.1)	13 (1.3)
Remote only	94 (10.9)	161 (15.8)	197 (20.0)	187 (19.2)
Did not work	90 (10.4)	337 (33.0)	569 (57.7)	650 (66.7)
Other ARI	(n = 1,266)	(n = 1,424)	(n = 1,362)	(n = 1,341)
Onsite only	984 (77.7)	719 (50.5)	419 (30.7)	422 (31.5)
Hybrid	47 (3.7)	49 (3.4)	31 (2.3)	30 (2.2)
Remote only	148 (11.7)	209 (14.7)	265 (19.5)	241 (18.0)
Did not work	87 (6.9)	447 (31.4)	647 (47.5)	648 (48.3)

*We excluded persons with influenza during Oct 2020–Jun 2022 (57, 65, 70, and 67 for day before, day 1, day 2, and day 3, respectively), persons with only remote work experience before illness onset (560, 676, 689, and 677 for day before, day 1, day 2, and day 3, respectively), and persons with missing work location (41, 74, 55, and 50 for day before, day 1, day 2, and day 3, respectively).
†p<0.05 (comparison of work location by test result for specified day).
‡p<0.001 (comparison of work location by test result for specified day).

Appendix Table 11. Likelihood of working onsite among adults who were scheduled to work, by PCR test result and reported fever, United States, 2018–2022*

Characteristic	Day 1 of illness (n = 4,929)	Day 2 of illness (n = 4,691)	Day 3 of illness (n = 4,588)
Prepandemic influenza seasons			
Influenza			
Fever	58.4 (415/711)	31.9 (224/702)‡	26.8 (185/691)†
No fever	64.0 (89/139)	45.9 (61/133)	34.9 (51/146)
Other ARI			
Fever	62.3 (538/864)	45.1 (362/802)§	42.9 (327/762)§
No fever	62.1 (497/801)	57.3 (421/735)	58.0 (409/705)
COVID-19 pandemic period			
COVID-19			
Fever	49.8 (309/620)	20.1 (121/601)†	10.9 (63/577)§
No fever	54.1 (207/383)	26.2 (97/370)	19.4 (73/377)
Other ARI			
Fever	49.9 (336/674)‡	29.0 (188/648)‡	28.1 (177/629)§
No fever	57.5 (424/737)	36.9 (258/700)	39.1 (274/701)

*Values represent % worked onsite (no. worked onsite / no. scheduled to work) except as indicated. Worked onsite represents onsite only or hybrid work location. We excluded persons with influenza during the COVID-19 pandemic period (65, 70, and 67 for day 1, day 2, and day 3, respectively), persons with only remote work experience before illness onset (676, 689, and 677 for day 1, day 2, and day 3, respectively), persons with missing work location (74, 55, and 50 for day 1, day 2, and day 3, respectively), and persons with missing information on fever (30, 29, and 31 for day 1, day 2, and day 3, respectively).
†p<0.05 (comparison of % worked onsite for specified illness and day).
‡p<0.01 (comparison of % worked onsite for specified illness and day).
§p<0.001 (comparison of % worked onsite for specified illness and day).

Appendix Table 12. Likelihood of working onsite among adults with COVID-19 illness who were scheduled to work, by day when COVID-19 positive PCR and at-home test results were available, United States, January 2022–June 2022*

Characteristic	Worked onsite, %
Scheduled to work on day 1 of COVID-19 illness†	
COVID-19 positive PCR result available on day 1 of illness	83.3 (5/6)
COVID-19 positive PCR result available after day 1 of illness	50.3 (173/344)
COVID-19 positive at-home result available on day 1 of illness	38.7 (12/31)
Excluding persons in the category above	51.4 (161/313)
Scheduled to work on day 2 of COVID-19 illness‡	
COVID-19 positive PCR result available on day 1 or 2 of illness	0.0 (0/56)#
COVID-19 positive PCR result available after day 2 of illness	18.6 (52/280)
COVID-19 positive at-home result available on day 1 or 2 of illness	11.1 (7/63)
Excluding persons in the category above	20.7 (45/217)
Scheduled to work on day 3 of COVID-19 illness§	
COVID-19 positive PCR result available on day 1, 2, or 3 of illness	3.5 (4/116)¶
COVID-19 positive PCR result available after day 3 of illness	12.0 (26/216)
COVID-19 positive at-home result available on day 1, 2, or 3 of illness	11.3 (7/62)
Excluding persons in the category above	12.3 (19/154)

*Values represent % worked onsite (no. worked onsite / no. scheduled to work). Worked onsite represents onsite only or hybrid work location. Analysis is based on persons with COVID-19 shown in Table 5 who enrolled in the study on or after January 15, 2022. Day of illness when COVID-19 positive result was available was computed by comparing the date of illness onset with the date that COVID-19 positive test result was available.
†Unknown when COVID-19 positive PCR result was available = 16 persons.
‡Unknown when COVID-19 positive PCR result was available = 19 persons.
§Unknown when COVID-19 positive PCR result was available = 18 persons.
¶p<0.01 (comparison of the two PCR result categories for specified day of illness).
#p<0.001 (comparison of the two PCR result categories for specified day of illness).

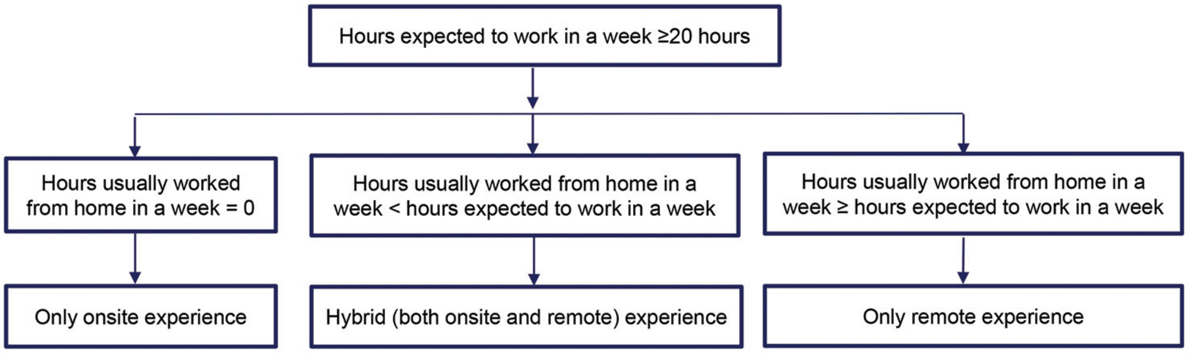

Appendix Figure 1. Algorithm to categorize work experience before illness onset, United States, 2018–2022. We excluded participants from analysis if hours expected to work in a week was <20 hours.

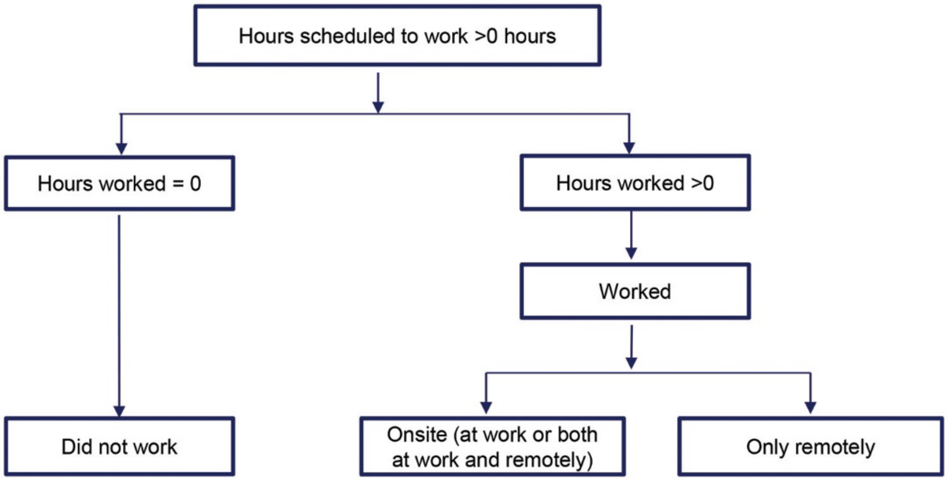

Appendix Figure 2. Algorithm to categorize work attendance on the day before illness onset and during the first 3 days of illness among persons who were scheduled to work, United States, 2018–2022. We categorized participants as not scheduled to work if hours scheduled to work was zero.

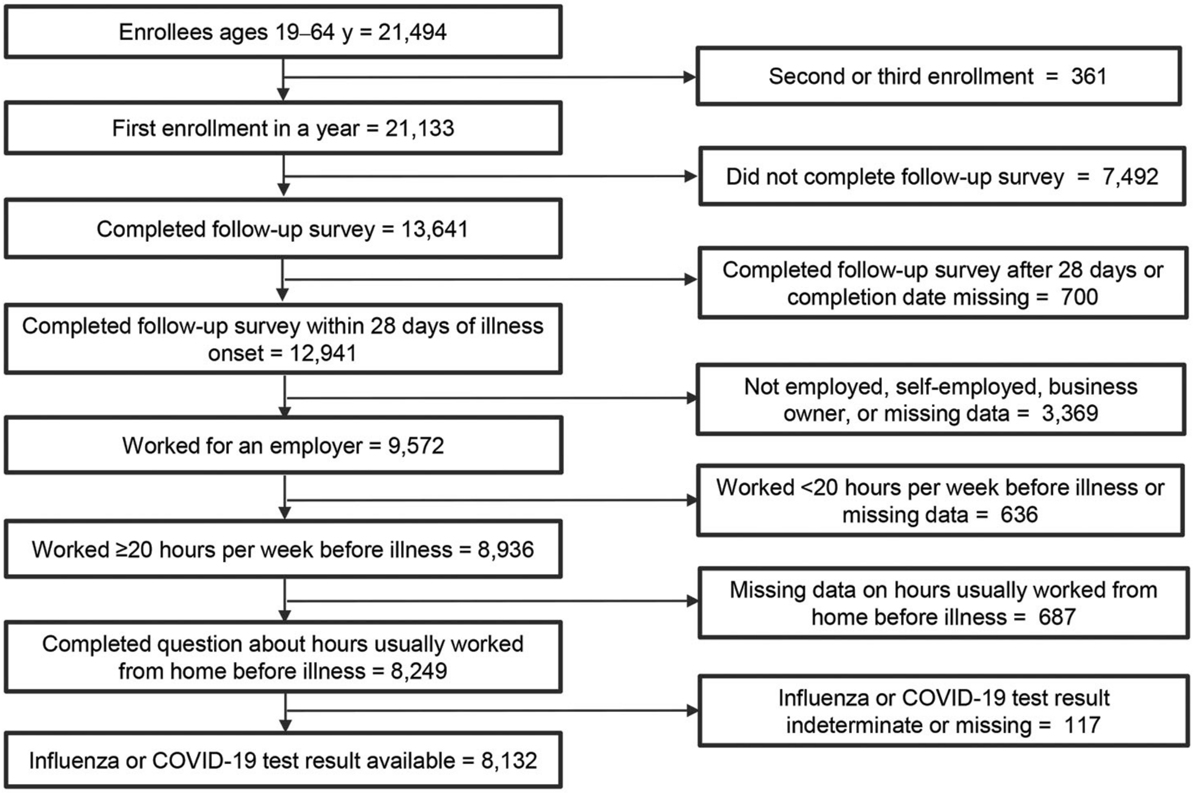

Appendix Figure 3. Assembly of study participants with influenza, COVID-19, or other acute respiratory illness, United States, 2018–2022.

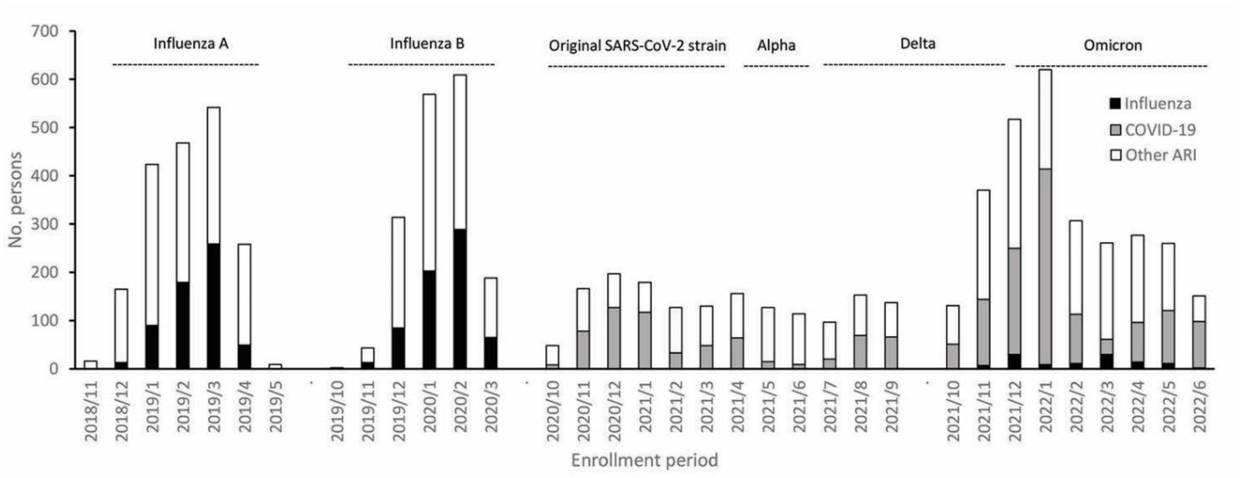

Appendix Figure 4. Period of enrollment of adults with influenza, COVID-19, or other acute respiratory illness (ARI) who were included in the analysis (n = 8,132), United States, 2018–2022. During November 2018–March 2020, there were 1,245 persons with influenza and 2,362 persons with other ARI. During October 2020–June 2022, there were 114 persons with influenza, 1,888 with COVID-19 (including seven persons who had both COVID-19 and influenza), and 2,523 with other ARI. The proportion of persons

with fever was 82.6% (1,121/1,358) for influenza, 61.4% (1,142/1,859) for COVID-19, and 49.6% (2,409/4,860) for other ARI ($p<0.001$) (information on fever was missing for 55 persons). The dominant influenza and SARS-CoV-2 strains and variants in the United States, which are shown at the top of the figure, represent periods during which a strain or variant comprised >50% of sequenced isolates (references: https://doi.org/10.1093/infdis/jiz543, http://dx.doi.org/10.15585/mmwr.mm6907a1, http://dx.doi.org/10.15585/mmwr.mm7116e1, http://dx.doi.org/10.15585/mmwr.mm7225a3).
